# Serological proteomic screening and evaluation of a recombinant egg antigen for the diagnosis of low-intensity *Schistosoma mansoni* infections in endemic area in Brazil

**DOI:** 10.1371/journal.pntd.0006974

**Published:** 2019-03-14

**Authors:** Vanessa Silva-Moraes, Lisa Marie Shollenberger, William Castro-Borges, Ana Lucia Teles Rabello, Donald A. Harn, Lia Carolina Soares Medeiros, Wander de Jesus Jeremias, Liliane Maria Vidal Siqueira, Caroline Stephane Salviano Pereira, Maria Luysa Camargos Pedrosa, Nathalie Bonatti Franco Almeida, Aureo Almeida, Jose Roberto Lambertucci, Nídia Francisca de Figueiredo Carneiro, Paulo Marcos Zech Coelho, Rafaella Fortini Queiroz Grenfell

**Affiliations:** 1 Biologia do *Schistosoma mansoni* e sua interação com o hospedeiro, Instituto René Rachou, Fundação Oswaldo Cruz, Belo Horizonte, Minas Gerais, Brazil; 2 Department of Infectious Diseases, College of Veterinary Medicine, University of Georgia, Athens, Georgia, United States of America; 3 Laboratório de Enzimologia e Proteomica, Universidade Federal de Ouro Preto, Ouro Preto, Minas Gerais, Brazil; 4 Grupo de Pesquisas Clínicas e Políticas Públicas em Doenças Infecciosas e Parasitárias, Instituto René Rachou, Fundação Oswaldo Cruz, Belo Horizonte, Minas Gerais, Brazil; 5 Laboratório de Biologia celular, Instituto Carlos Chagas, Fundação Oswaldo Cruz, Curitiba, Paraná, Brazil; 6 Faculdade de Medicina, Universidade Federal de Minas Gerais, Belo Horizonte, Brazil; 7 Faculdade de Medicina, Universidade Estadual de Montes Claros, Montes Claros, Brazil; Queen’s University Belfast, UNITED KINGDOM

## Abstract

**Background:**

Despite decades of use of control programs, schistosomiasis remains a global public health problem. To further reduce prevalence and intensity of infection, or to achieve the goal of elimination in low-endemic areas, there needs to be better diagnostic tools to detect low-intensity infections in low-endemic areas in Brazil. The rationale for development of new diagnostic tools is that the current standard test Kato-Katz (KK) is not sensitive enough to detect low-intensity infections in low-endemic areas. In order to develop new diagnostic tools, we employed a proteomics approach to identify biomarkers associated with schistosome-specific immune responses in hopes of developing sensitive and specific new methods for immunodiagnosis.

**Methods and findings:**

Immunoproteomic analyses were performed on egg extracts of *Schistosoma mansoni* using pooled sera from infected or non-infected individuals from a low-endemic area of Brazil. Cross reactivity with other soil-transmitted helminths (STH) was determined using pooled sera from individuals uniquely infected with different helminths. Using this approach, we identified 23 targets recognized by schistosome acute and chronic sera samples. To identify immunoreactive targets that were likely glycan epitopes, we compared these targets to the immunoreactivity of spots treated with sodium metaperiodate oxidation of egg extract. This treatment yielded 12/23 spots maintaining immunoreactivity, suggesting that they were protein epitopes. From these 12 spots, 11 spots cross-reacted with sera from individuals infected with other STH and 10 spots cross-reacted with the negative control group. Spot number 5 was exclusively immunoreactive with sera from *S*. *mansoni*-infected groups in native and deglycosylated conditions and corresponds to Major Egg Antigen (MEA). We expressed MEA as a recombinant protein and showed a similar recognition pattern to that of the native protein via western blot. IgG-ELISA gave a sensitivity of 87.10% and specificity of 89.09% represented by area under the ROC curve of 0.95. IgG-ELISA performed better than the conventional KK (2 slides), identifying 56/64 cases harboring 1–10 eggs per gram of feces that were undiagnosed by KK parasitological technique.

**Conclusions:**

The serological proteome approach was able to identify a new diagnostic candidate. The recombinant egg antigen provided good performance in IgG-ELISA to detect individuals with extreme low-intensity infections (1 egg per gram of feces). Therefore, the IgG-ELISA using this newly identified recombinant MEA can be a useful tool combined with other techniques in low-endemic areas to determine the true prevalence of schistosome infection that is underestimated by the KK method. Further, to overcome the complexity of ELISA in the field, a second generation of antibody-based rapid diagnostic tests (RDT) can be developed.

## Author summary

Schistosomiasis remains a serious global public health problem. Detecting parasite eggs in patient stool samples using the KK method is the standard diagnostic recommended by the World Health Organization (WHO) for infection by *S*. *mansoni*. As a result of intensive control strategies, many previously high-endemic areas are now considered low-endemic areas and the KK method does not function well in low-endemic areas and therefore cannot be considered the gold standard. Thus, a new emphasis on strategies to accurately diagnose low-intensity infections was outlined in a plan from the WHO focusing on elimination of disease as a public health problem. Successful diagnoses and treatment of infected individuals may result in eradication of low-burden transmitters and consequently contribute to interruption of disease transmission. In this regard, immunological techniques have proven to be more sensitive and promising for identifying low-intensity infections where KK may be negative. The identification of antigens is the initial step for developing new immunodiagnostic assays. In this study, we used sets of pooled human sera samples from controls with acute and chronic infections to identify new target antigens via proteomic screening. Using these approaches, we initially identified 12 different egg proteins in *S*. *mansoni*-infected individuals (acute and chronic phase). A single antigen, identified as MEA, was shown to be highly specific as this antigen was not recognized by sera from negative patients or patients infected with other STH. The recombinant MEA protein functioned in an ELISA as a highly sensitive and specific antigen to detect patient IgG-antibodies. Recombinant MEA performed significantly better to detect low-intensity infections (1 egg per gram of feces) than the KK method using 2 slides. Therefore, we were able to use a proteomic screening approach to identify a potential new candidate antigen for development of far more sensitive diagnostic assays. Further diagnostic assays employing the MEA could be useful tools on their own or in combination with other methods for diagnosis of schistosome infection in populations living in extreme low-intensity endemic areas of Brazil.

## Introduction

Schistosomiasis remains as a major worldwide public health problem. Since it is a disease of poverty and limited sanitary facilities, the disease has proved difficult to control for centuries [1]. Schistosomiasis afflicts low-income populations in tropical and subtropical regions with varying levels of morbidity and mortality and has a significant socioeconomic impact [2]. Estimates suggest that approximately 290 million people are affected in 78 countries around the world, especially in Sub-Saharan Africa, Asia, and South America [3]. Brazil has the highest burden of disease in the Americas and infection is caused by *S*. *mansoni* [4].

During the past 40 years, Brazil has developed an extensive history regarding the fight against schistosomiasis. Integrated control measures, such as investments in basic sanitation and hygiene, improvement in the population’s income levels and quality of life, and chemotherapy have had considerable success in terms of reducing prevalence, transmission and parasite loads [5]. The prevalence in Brazil was estimated at 1% by the National Schistosomiasis and Soil-transmitted Helminth Infection Survey (INPEG), conducted between 2010 and 2015 [5]. Despite this significant reduction in prevalence, the disease has acquired a new epidemiological profile. Currently, Brazil has multiple endemic areas where chronically infected patients have low-intensity infections (number of eggs per gram of feces, EPG, <100) [5–8]. The continuous distribution of disease remains mainly in the Northeast and Southeast regions of the country. Focal transmission, followed by acute infection, has also been reported as a result of migration of infected individuals (rural tourism and urbanization) [5, 9–11].

In this new epidemiological scenario, infected individuals are very unlikely to be detected with routine parasitological methods. Since praziquantel (PZQ) mass drug administration is not conducted in Brazil, the main strategy to control and eliminate the disease is diagnosis and treatment of active cases [4, 12]. As recommended by WHO, diagnosis of schistosomiasis continues to be detection of schistosome eggs in stools by microscopic examination using the KK technique [13]. The KK method is low-cost and suitable for detection of medium and high-intensity infections, i.e. > 100 EPG. However, it has poor sensitivity for detection of low-intensity infections that are seen in residents living in low-endemic areas (<10% prevalence, <100 EPG) [6–8, 14, 15]. As consequence, many true positive individuals are missed, generating significant underestimation of prevalence and shortcomings on control programs. Previous studies in Brazil demonstrated that prevalence has been underestimated by a factor of 2–4, due to the inability of the KK method to detect low-intensity infections [6–8, 16, 17]. The failure to diagnose infected individuals contributes to continuation of *S*. *mansoni* infection, followed by contamination of the environment and maintenance of transmission. If the goal of elimination is a priority for the WHO [1, 9], new and more sensitive methods need to be applied to achieve it.

The development of new methods that have the ability to accurately diagnose low-intensity infections was outlined in the WHO’s plans focusing on elimination of schistosomiasis as a public health problem [9, 18, 19]. In this regard, molecular and immunological techniques have proven to be more sensitive and promising for identifying infected individuals that are negative by KK coproscopy results [8, 16, 17, 20–22]. Significant progress has been seen in the development of antigen-based rapid diagnostic tests (RDT), as their assembly is user-friendly in the field. The immunochromatographic point-of-care (POC) test that detects circulating cathodic antigen (CCA) in urine has been commercially available since 2008 [23, 24]. Although POC-CCA has been suggested to be a suitable substitute for KK in *S*. *mansoni* prevalence mapping [24–27], its performance is still debatable in low-endemic areas [28–30]. Most studies validating POC-CCA were conducted in Africa, whereas few (10) studies were conducted in Brazil, which has a significantly different prevalence and morbidity profile. In contrast to Africa where low-intensity infections range from 1–100 EPG, most infections in Brazil are denoted as < 25 EPG [6, 7, 14, 22, 29, 31–35]. Furthermore, the KK method was used as a reference standard during the validation of POC-CCA in Africa. However, it is not sensitive enough to serve as a gold standard [28].

Indirect techniques based on detection of antibodies have high sensitivity in detecting low-intensity infections and are capable of identifying loads of 1 EPG [17, 21, 36–41]. In endemic settings, antibody-based methods present low specificity and are not indicated as single use tests. However, their use as screening tool combined with parasitological evaluations has decreased false-negative cases seen when only utilizing 2 KK slides in endemic settings [16, 21, 39, 40]. The indirect diagnostics can also detect pre-patent infections from individuals returning from schistosomiasis endemic areas. As antibodies to the parasite develop during the first weeks of infection, they can be detected before eggs are produced and released in the feces. In clinical practice, positive serology in KK negative people from non-endemic countries is usually sufficient to prescribe treatment with PZQ [10, 42, 43].

Antibody-based methods have been re-evaluated in order to improve detection of *S*. *mansoni* infection in endemic populations. As an alternative to enhance the specificity of assay, some studies focus on detection of specific antigens [44–47]. Crude antigens, such as soluble eggs antigens (SEA) and worm antigens (SWAP), are frequently used, but they can exhibit low-sensitivity and cross-reactivity with different helminths [48, 49]. Ideally, antibody detection should be performed using a specific, purified schistosome component or a schistosome-derived recombinant protein as the immunodiagnostic target. A combination of proteomic and serological analyses have served as promising experimental approaches for screening new biomarkers in the diagnostic field [50–52]. However, there is a limited number of serological-proteomic studies involving *Schistosoma spp*. and most of them are related to searching for vaccine candidates using animal models [52–57]. Only one immunoproteomic analysis related to *S*. *mansoni* and human samples has been performed to date, but it focuses on the search for vaccine candidates [57].

In the present work, we adopted immunoproteomic analysis to identify a new antigen candidate to be applied in schistosomiasis diagnosis. As antibodies against schistosome eggs have been considered useful antigens for the diagnosis of schistosomiasis [37, 43, 58], we screened soluble egg extracts (SEE) by two-dimensional western blotting (2D-WB). To achieve higher specificity, we compared native SEE extracts to those oxidized by sodium metaperiodate (SMP), in order to exclude antigens whose epito pes were glycan-based, since they denote high cross-reactivity among different helminths. Moreover, we analyzed the potential of the new target (MEA) as recombinant antigen for detecting individuals with low-intensity infection by ELISA.

## Methods

### Ethics statement

The present study was approved by the Ethics Committee of the Research Center Rene Rachou/Fiocruz under the following number: 893.582 11/2014 and by the National Brazilian Ethical Board under the following number: 14886. Before any research activities, the local health authorities were contacted and they agreed to collaborate with the researchers from different institutions. All enrolled participants were required to sign an informed consent form. Parents or legal guardians signed the informed consent when minors less than 18-years old were involved. When the parasitological results were positive, the relevant individuals were informed and received free oral treatment at the local health clinic. Schistosomiasis: PZQ (50 mg/kg for adults and 60 mg/kg for children); intestinal helminths: albendazole (400 mg); protozoan parasites: metronidazole (250 mg/2x/ 5 days).

All procedures involving animals were conducted in compliance with the Manual for the Use of Animals/FIOCRUZ and approved by the Ethics Committee on the Use of Experimental Animal (CEUA–FIOCRUZ) license number LW-31/15.

### Human samples

#### Chronic individuals

Samples from chronic individuals were obtained from Pedra Preta, Tabuas and Estreito de Miralta (491 residents, 243 women/250 men, 1–94 years old), rural communities localized in city of Montes Claros, state of Minas Gerais, Brazil [6, 8, 29, 31]. The study was performed from 2009–2014, including 491 residents (243 women/250 men, 1–94 years old). The rural region is a schistosomiasis low-endemic area where most individuals have low-intensity infections (< 100 EPG). The communities were selected based on environmental conditions, the presence of *Biomphalaria glabrata* snails (the intermediate host of *S*. *mansoni*), previously reported prevalence, and low migration rate.

All individuals were subject to parasitological evaluation to determine the diagnosis of schistosomiasis as well as other STH. Each resident provided a single sample that was used to make 24 KK slides (24 x 41.7 mg = 1 gram) (Helm-Test, Biomanguinhos, FIOCRUZ, Brazil) [13] and 2 procedures of Saline Gradient (SG) technique (2 x 500 mg = 1 gram) [59]. This protocol has been used previously in low-endemic areas and is considered a reference standard [6, 29, 31]. Results were reported as EPG of feces for both methods. Participants infected with *S*. *mansoni* and/or other helminths were treated with PZQ (60 mg/kg for children and 50 mg/kg for adults) and albendazole (400 mg), respectively, in single oral dose, as recommended by the Brazilian Health Ministry. Positive patients were followed up 30, 90, and 180 days post-treatment and the same baseline procedure was performed. Individuals testing positive post-treatment were retreated as needed. Individual serum samples from all participating individuals were obtained after centrifugation of blood samples at 3000g for 5 min and were maintained at -20°C until use.

Sera samples were selected and grouped according to 1) individuals positive for *S*. *mansoni*, but not infected with other STH, 2) individuals negative for *S*. *mansoni* and other STH at 180 days post-treatment, and 3) individuals positive for STH (*Ascaris lumbricoides*, *Trichuris trichiura* and *Ancylostoma*), but not *S*. *mansoni*. Fifteen sera samples for each group 1, 2 and 3 were pooled to perform the immunoproteomic analysis. Ninety-three sera samples from group 1 (41 women/52 men, 5–80 years old) were used for standardization of the IgG-ELISA. Eighty sera samples (36 women/44 men, 5–82 years old) from individuals negative for *S*. *mansoni* at baseline were also evaluated by IgG-ELISA.

#### Acute individuals

Fifty tourists (19 women/31 men, 4–75 years old) bathed in a swimming pool supplied by a brook in the outskirts of São João del Rei, a historical city in the state of Minas Gerais, Brazil from December 2009 to March 2010 as previously described [10]. Two months later, a patient was diagnosed with schistosomal myeloradiculopathy and he reported that other tourists had symptoms consistent with acute schistosomiasis. All participants submitted to clinical, laboratory, and ultrasound examinations, and the outbreak was confirmed. Authorities investigated the species of snail surrounding the area and *Biomphalaria glabrata* was the only species found. It was determined that transmission occurred because of in-migration of infected workers who were hired to build houses. This caused the non-endemic area to become a new focus of transmission. Diagnosis of acute *S*. *mansoni* schistosomiasis was based on epidemiologic data (recent contact with stream water in an endemic area), clinical data (i.e. cercarial dermatitis, acute enterocolitis, fever, cough, malaise, paraplegia, pulmonary involvement, hepatomegaly and/or splenomegaly), laboratory assays (i.e. eosinophilia, IgG antibodies against soluble worm antigens, eggs in the stool or in rectal biopsy fragments), and imaging techniques (i.e. ultrasound with liver and/or spleen enlargement and lymph node adenopathy, magnetic resonance showing spinal cord injury). To be considered as having acute schistosomiasis in the present study, the participants had to have one or more of the symptoms/signs described above, evidence of an infection (parasitological or serologic), and reported contact with contaminated waters of the swimming pool. All 50 individuals fulfilled the criteria for the case definition of acute *S*. *mansoni* schistosomiasis and were treated with PZQ (60 mg/kg for children and 50 mg/kg for adults). From 50 individuals, 19 presented eggs in the feces after KK examination (2 slides for each 2 stool samples) performed between 3 and 4 months after the date of contact with contaminated water. In this present study, 15 acute sera samples with egg-positive results were pooled for immunoproteomic analysis and classified as group 4.

#### Healthy individuals

Fifty-five healthy individuals (35 women/20 men, 21–70 years old) living in a non-endemic area in Belo Horizonte, state of Minas Gerais, were selected as donors to be used as our negative control group (group 5). They were interviewed and had no medical history of previous schistosomiasis. Parasitological examination was performed by KK and SG as previously described [6]. Serological reactivity to SEA and SWAP was performed by IgG-ELISA in the Reference Center for Schistosomiasis as previously described [37]. Patients with eggs in the feces and reactive for both IgG-ELISA assays were removed from the healthy group. In this present study, 15 sera samples were pooled for immunoproteomic analysis and all 55 sera samples from group 5 were used for standardization of the IgG-ELISA.

### Immunoproteomics analysis

#### Preparation of soluble egg extract

The preparation of SEE was performed as Ashton et al. (2001) with modifications [60]. The *S*. *mansoni* LE strain, isolated in Brazil and maintained according to Pellegrino and Katz at the Rene Rachou Research Center, was used to infect BALB/c mice female [61]. The mice were infected by the subcutaneous route with 100 cercariae and sacrificed after 45 days. Their livers were removed, homogenized and digested with 0.02% trypsin from porcine pancreas (type IX-S, Sigma-Aldrich) for 3 h at 37°C. After incubation, the livers were sieved and the eggs were collected by sedimentation and cleaned by washing 6 times in Phosphate-Buffered Saline (PBS). Cleaned eggs were re-suspended in 1 mL of Tris-Buffered Saline (TBS) supplemented with protease inhibitor cocktail (Sigma-Aldrich) and 1% dithiothreitol (DTT). The suspension was sonicated using six 10 sec pulses on full power with 1 min on ice between each sonication. Sonicated suspension was centrifuged at 100,000 g for 60 min and the supernatant collected. Protein concentration of SEE was measured by the Bicinchoninic Acid Assay (BCA) (ThermoScientific) and the quality of the extract was verified by SDS-PAGE 12%. Next, acetone precipitation was performed and the pellet was solubilized in rehydration buffer (7 M Urea, 2 M Thiourea, 2% 3-3-Cholamidopropyl-dimethylammoniopropane-sulfonate (CHAPS), 0.002% bromophenol blue) and stored at -70°C until use.

#### Two-dimensional-polyacrylamide-gel-electrophoresis (2D-PAGE)

Sixty μg of protein extract was used for 2D-PAGE to be stained in gel and 45 μg of extract for 2D-PAGE to be used for Western blot. SEE proteins solubilized in rehydration buffer were supplemented with 1% DTT and 0.8% ampholyte 3–10 buffer (Bio-Lyte, Bio-Rad) and submitted to first dimension. Samples were loaded onto 7 cm immobilized pH gradient (IPG) strips, 3–10 pH ranges (Immobiline DryStrip Gels, GE Healthcare) for isoelectric focusing (IEF). IEF was conducted in Ettan IPGphor 3 (GE Healthcare) at 20°C and 50 μA/strip under the following conditions: passive in-gel rehydration at 50 V for 12 hs and focalization at 500 V for 30 min, followed by 1,000 V for 30 min and 8,000 V for 3 hs. Focused proteins were reduced and then alkylated using 1% DTT and 4% iodoacetamide, respectively, in equilibration solution (6 M urea, 75 mM Tris-HCl pH 8.8, 30% glycerol, 2% SDS, 0.002% bromophenol blue) for 15 min each at room temperature (RT). For the second dimension, IPG strips and molecular weight standards were then placed on top of 12% SDS-PAGE gels and sealed with 1% agarose. Electrophoretic protein separation was performed using the Mini-Protean III (Bio-Rad) at 20 mA/gel, for approximately 6 h. Gels were fixed in 2% v/v orthophosphoric acid/30% v/v ethanol solution overnight, then washed 3 × 10 min with 2% v/v orthophosphoric acid. Gels were stained with 2% v/v orthophosphoric acid/18% v/v ethanol/15% w/v ammonium sulfate/0.002% w/v Colloidal Coomassie Blue G-250 (Sigma-Aldrich) solution for 48 h. Gels were destained in 20% v/v ethanol for 5 min. For each experiment, three 2D-PAGEs were performed in parallel, one for Western blotting with native extract, another for western blotting with deglycosylated extract and another for stain and spot excision for protein identification.

#### Two-dimensional western blotting (2D-WB)

Proteins in 2D-PAGE were electrophoretically transferred to PVDF membrane 0.2 μm (GE Healthcare) using a Mini-Trans-Blot (Bio-Rad) at 100 V (2–3 mA cm2) for 2 h at 4°C with transfer buffer (25 mM Tris-Base, 192 mM glycine, 20% methanol). Post-transfer, PVDF membranes were stained with Ponceau for 10 min and quickly washed in water. Membranes were then blocked with TBS (20 mM Tris-HCl, 500 mM NaCl, pH 7.5) containing 0.05% Tween-20 and 5% skim milk (TBS-T/5% milk) at 4°C for 16 h. Membranes were washed five times, 5 min/wash, in TBS-T. To alter glycan structures on the membranes, we used the method of Woodward et al. (1985) [62]. Membranes were incubated in 10 mM Sodium Metaperiodate (SMP) solution in 50 mM acetate buffer, pH 4.5, at RT for 1 h in the dark. Membranes were then washed with acetate buffer for 10 min, then incubated in 50 mM sodium borohydride in PBS for 30 min at RT. After five, 5-min washes with TBS-T, membranes were individually incubated in pooled human sera. The native membranes and SMP-treated membranes were incubated separately for 2 h with each pool of group 1, 2, 3, 4 and 5 sera diluted 1:600 in TBS-T/3% milk and INF-AC sera diluted 1:1200 in TBS-T/3% sera. After five, 5-min washes with TBS-T, the membranes were incubated with anti-human IgG peroxidase conjugated antibody (A0170, Sigma-Aldrich), diluted 1:100,000 in TBS-T/3% milk at RT for 1 h. After five, 5-min washes with TBS-T and one 10-min wash in TBS, the immunoreactive proteins were developed using ECL Plus Western Blotting Detection System (GE Healthcare) and images captured using chemiluminescence detection in ImageQuant LAS 4000 (GE Healthcare). The 2D-WB experiments were performed in duplicate.

#### In gel-digestion

The 2D-WB and its corresponding Coomassie stained 2D-PAGE were overlapped using software Photoshop (Adobe Systems Incorporated) and spots identified in duplicate experiments were selected for identification. The antigenic protein spots were manually and individually excised from the corresponding 2D-PAGE for mass spectrometry (MS) identification. Selected spots were destained in 40% ethanol/7% acetic acid at 37°C until clear. Gel pieces were then washed in ultrapure water and reduced in 50 mM DTT at 65°C for 30 min and then alkylated in 100 mM iodoacetamide, at RT for 1 h. Gel pieces were then washed in 20 mM ammonium bicarbonate (AB)/50% acetonitrile (ACN) for 3 x 20 min each and fully dried using Speed Vac Concentrator Plus (Eppendorf). Gel slices were rehydrated in 15 μL of the digestion buffer containing 0.01 μg/μL of Sequencing Grade Modified Trypsin (Promega) in 20 mM AB for 20 min. Excess trypsin was removed and additional 40 μL of 20 mM AB added. Trypsinolysis was performed for 48 h at 37 °C. Then digestion supernatants transferred to clean tubes and 50 μL of 0.1% trifluoroacetic acid (TFA)/50% ACN were added to gel slices for 30 min. The supernatants from both tubes containing the tryptic peptides were pooled, dried by speed vacuum, and re-suspended in 10 μL of 0.1% TFA. The peptides were desalted in reverse phase micro-columns Zip Tip C18 (Millipore), according to manufacture instructions. Peptides were dried again and re-suspended in 20 μL of 0.1% TFA for liquid chromatography–mass spectrometry (LC-MS) analysis.

#### Protein identification by mass spectrometry

Digestion products were analyzed by liquid chromatography–mass spectrometry (LC-MS) on a Q-Exactive hybrid quadrupole-orbitrap mass spectrometer (Thermo Scientific). Four μl of peptide samples were injected into a nano UHPLC instrument (Dionex UltiMate 3000, Thermo Scientific) through a trapping system (Acclaim PepMap100, 100 um x 2 cm, C18, 5 um, 100 A, Thermo Scientific) for 3 min using 98% water / 2% ACN with 1% TFA as solvent and subsequently directed into a capillary column (Acclaim PepMap100, 75 um x 25 cm, C18, 3 um, 100 A, Thermo Scientific). Reverse-phase separation of peptides was performed at 40°C in a gradient of solvent A (water, 0.1% formic acid) and B (80% ACN / 20% water, 0.1% formic acid), at a flow rate of 300 nL/min. Peptides were sequentially eluted over a gradient spanning from 3.2% to 12% ACN over 2 min and from 12% to 44% ACN over an additional 15 min. Peptide ions were detected using positive mode through data dependent analysis. Resolution for precursor ions (MS1) was set to 70,000 (FWHM at 200 *m/z*) with an automatic gain control target of 3e^6^, maximum injection time of 100ms, scanning over 300–2000 *m/z*. The Top12 most intense precursor ions of each MS1 mass spectra were individually isolated with a 2.0 Th window for activation via higher-energy collisional dissociation (HCD) with normalized energy of 30 V. Only peptides exhibiting charge states of +2, +3, +4 and +5 were selected. Automatic gain control target was set to 5e^5^ (minimum accumulation of 3.3e^3^) with maximum injection time of 150 ms. Dynamic exclusion of 40 sec was active.

Spectral data was submitted to Proteome Discoverer v.1.4 (Thermo) for database search using SEQUEST HT against 10.779 *S*. *mansoni* protein sequences (5.136.273 residues). Search parameters included cysteine carbamidomethylation as a fixed modification, methionine oxidation and protein N-terminal acetylation as variable modifications, up to one trypsin missed cleavage site, error tolerance of 10 ppm for precursor and 0.1 Da for product ions. A quality filter was applied to keep False Discovery Rate (FDR) < 0.1. The average area for the 3 most intense peptides was used to infer protein abundance. This was particularly important when more than one protein identity was assigned to the same gel spot. Only proteins identified with at least 2 unique peptides were considered in this study.

### Production of recombinant protein

#### Cloning, expression and purification of MEA

The recombinant *S*. *mansoni* MEA (rMEA) was produced by Gateway cloning technology (Invitrogen). First, the coding region from the gene of interest was obtained by PCR amplification using cDNA from *S*. *mansoni* eggs as template (BEI Resources, Catalog no. NR-49421, U.S. Government property). Based on the nucleotide sequence (Smp_049250.1, GeneDB) the primers were designed: forward (5’-ATGTCTGGTGGGAAACAACATAACGCA-3’) and reverse (5’-CTAGTGAGTAATCGCATGTTGCTTCTCCAATG-3’). PCR amplification included DNA polymerase buffer 20 μL, 10 mM dNTP mixture 5 μL, 100 μM forward primer 1.5 μL, 100 μM reverse primer 1.5 μL, 50 ng cDNA, Q5 High-Fidelity DNA polymerase 1 μL (New England Biolabs), RNase-free dH_2_O to 100 μL final volume. Secondly, the purified PCR product was used as template for the following PCR amplification which involved DNA fragments flanked by attB sites. The primers were designed with sites attB incorporated and underlined in fusion with N-terminal histidine tag following the Gateway manufacturer’s instructions [63]: forward (5’-GGGGACAAGTTTGTACAAAAAAGCAGGCTTCGAAGGAGATAGAATGTCTGGTGGGAAACAACATAACGC-3’) and reverse (5’-GGGGACCACTTTGTACAAGAAAGCTGGGTCCTAGTGAGTAATCGCATGTTGC-3’). The PCR product with flanked attB sites were inserted into pDONR221 vector by BP recombination reaction at 25°C for 16 h yielding the entry clone. Next, the entry clone containing the gene of interest flanked by the attL sites was integrated into the destination vector pEXP1-DEST by LR recombination reaction at 25°C for 16 h, producing the final expression clone. After each recombination reaction, the clones were transformed into subcloning competent DH5α *Escherichia coli* (New England Biolabs) by heat shock. The positive colonies were selected by PCR and grown in Luria Bertani broth supplemented with 100 μg/mL ampicillin (LB-Amp). The vector constructions were purified by QIAprep Spin Miniprep Kit (Qiagen) and the DNA sequencing verified using the M13 primers for entry clone and T7 terminator primers for expression clone primers (Eurofins Genomics). The expression clone was transformed into competent BL21 (DE3) *E*. *coli* (Novagen) and grown in LB-Amp at 37°C for 12 h. The culture was diluted 100-fold in LB-Amp until achieving an absorbance of 0.6 in 600 nm. Protein synthesis was induced by addition of 1 mM isopropyl β-D-thiogalactoside (IPTG) at 37°C for 4 h. Cells were then harvested by centrifugation and re-suspended in 40 mL of lysis buffer (50 mM Tris, 0.5 M NaCl, 0.2 mM EDTA, 3% sucrose, 1% Triton-X and 10 mM imidazole). Subsequently, the cells were submitted to three 30 s-cycles of sonication and centrifuged at 5400 g for 20 min. The protein was purified by affinity chromatography on Ni-NTA column (HisPur Ni-NTA Spin Columns, Thermo) under native conditions (imidazole: binding 10 mM, washing 3 x 25 mM and 1 x 100 mM, elution 500 mM). The purification of rMEA was verified by SDS-PAGE and western blotting using an anti-histidine tag. Fractions containing rMEA were dialyzed against PBS pH 7.0 and concentrated using 30 kDa centrifugal tubes (Millipore). The recombinant proteins were quantified using BCA method and send to LC-MS to analysis by Shotgun.

#### Western blotting analyzes

Two, three and seven μg of rMEA were transferred onto 0.2 μm PVDF membrane lanes after electrophoresis. The membrane was blocked at 4°C for 16 h in TBS-T/3% skim milk. Subsequently *S*. *mansoni* positive chronic sera (group 1) and negative control sera (group 5) were added at 1:800 dilution and incubated at RT for 2 h. After five 5-min washes with TBS-T, the membrane was incubated with anti-human IgG peroxidase conjugated antibody diluted 1:80,000 in TBS-T/3% milk at RT for 1 h. The membrane was washed one more time and revealed as 2D-WB.

### Application of rMEA in immunodiagnostic assay

Recombinant antigen rMEA was evaluated for the ability to diagnose *S*. *mansoni* infection by antigen-specific IgG ELISA (rMEA-IgG-ELISA). Optimization of the protocol and dilution of reagents were determined by titration. Flat bottom plates (Maxisorp NUNC) were coated 100 μl/well with rMEA 1μg/mL in 0.05 M carbonate bicarbonate buffer pH 9.6 and incubated at 4°C for 16 h. The plates were washed six times in PBS with 0.05% Tween 20 (PBS-T) and blocked by addition of 300 μl/well of 2.5% skim milk in PBS-T at 37°C for 2 h. After additional washing, 100 μl/well of individual serum diluted 1:100 in PBS was added to the plate in duplicate and incubated at RT for 2 h. The plates were washed, and peroxidase conjugated anti-human IgG antibody was then added to wells at a dilution of 1:60,000 in PBS-T at RT for 1 h. After more washes, plates were developed using 3, 3', 5, 5'-tetramethylbenzidine (TMB, Sigma). The reaction was stopped after 10 min of incubation in the dark with 50 μL of sulfuric acid. The optical density (OD) was determined by an automatic ELISA reader (Multiskan, Thermo Scientific), using a filter at 450 nm.

### Statistical analysis

Analyses were performed using Open Epi, version 3.03 and GraphPad Prism, version 5.0. In order to evaluate the performance of rMEA-IgG-ELISA, a reference standard was established, which included all positive results (visible eggs) from any of the parasitological methods used (KK and SG). Normal distribution of the data was verified by the Shapiro-Wilk test. To compare the means for non-normal distribution, the Mann-Whitney test was used with a p-value ≤ 0.05 considered significant. Receptor Operating Characteristic curves (ROC curves) were used to calculate area under curve (AUC), sensitivity, specificity and the cut-off points between positive (group 1) and negative groups (group 5). The AUC indicates the probability of accurately identifying true positives, where one could distinguish between non-informative (AUC = 0.5), less accurate (0.5<AUC≤ 0.7), moderately accurate (0.7<AUC≤ 0.9), highly accurate (0.9<AUC<1) and perfect tests (AUC = 1) [64]. Positive predictive values (PPV), Negative Predictive Values (NPV) and overall accuracy (ACC) was determined by the following formula: PPV = number of true positives/(number of true positives + number of false positives); NPV = number of true negatives/(number of true negatives + number of false negatives) and ACC = (number of true positives + number of true negatives)/ (number of true positives + true negatives + number of false positives + number of false negatives).

The McNemar’s test was used to analyze categorical variables. To evaluate the degree of concordance between the different methods, the kappa index (κ) followed the categorization for Landis and Koch (1972): <0 poor, 0.00–0.20 slight, 0.21–0.40 fair, 0.41–0.60 moderate, 0.61–0.80 substantial and 0.81–1.00 almost perfect. The relationship between the intensity of infection (EPG) determined by parasitological tests and the IgG-ELISA (OD) was examined by the Spearman correlation test.

## Results

### MEA was the single protein recognized specifically by sera from *S*. *mansoni* positive individuals

**Fig 1:**
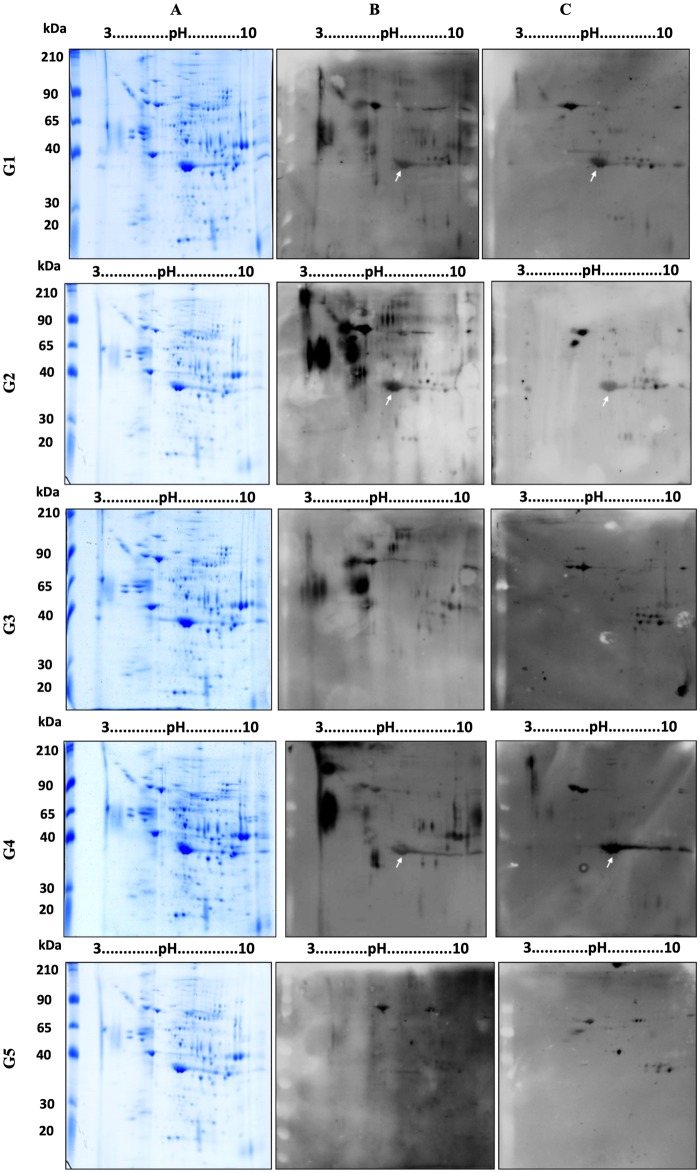
Two-dimensional analysis using *Schistosoma mansoni* egg extract and pooled sera from infected and non-infected individuals. A) 2D-PAGE of native SEE in 7 cm, pH 3–10 strip and stained by Coomassie G-250. B) Corresponding 2D-WB with pooled human sera and C) 2D-WB post membrane treatment with 10 mM of SMP. Sera from group 1, 2, 3 and 5 was added at 1:600 dilution. The sera from group 4 was added at 1:1200 dilution. The anti-human IgG peroxidase conjugate was added at 1:100,000. The white arrows indicate spot 5 corresponding to Major Egg Antigen. G1: *S*. *mansoni* chronic sera; G2: *S*. *mansoni* chronic sera after 180 days post treatment; G3: STH-positive sera (*Ascaris lumbricoides*, *Trichuris trichiura* and *Ancylostoma*); G4: *S*. *mansoni* acute sera, G5: negative sera from health donors.

The 2D-PAGE provided good resolution of spots in pH range with minimal streaking. In order to identify the antigens recognized by antibodies in pooled sera, a corresponding 2D-PAGE was performed in parallel so that WB (native and SMP-oxidized) could be performed to exclude any variation that might arise from the use of different antigen preparations (Fig 1).

**Fig 2:**
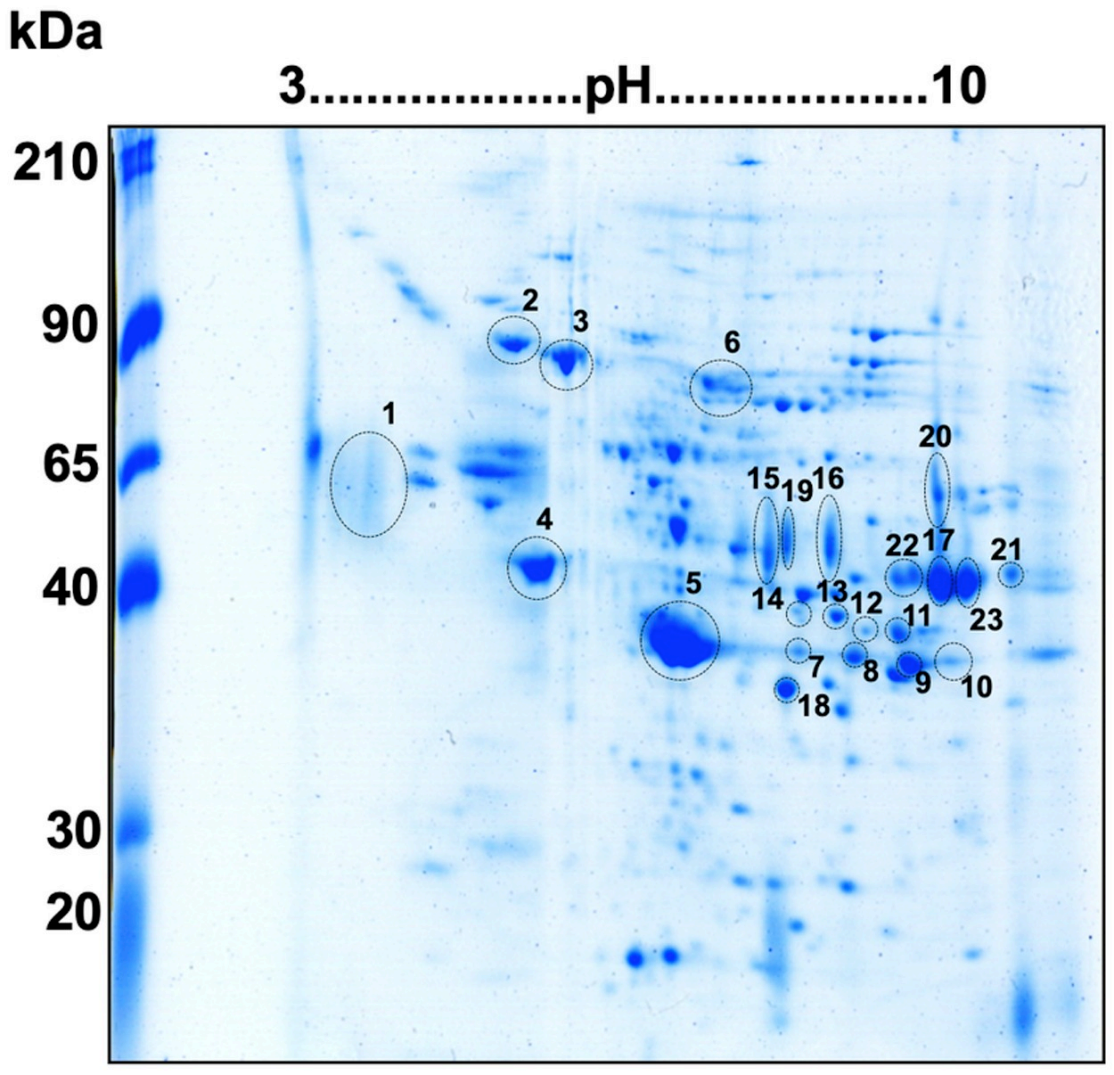
Coomassie blue-stained 2D-PAGE showing spots matched to the 2D-WB. Proteins from SEE were resolved in 7 cm, pH 3–10 strip and stained by Coomassie G-250. Immunoreactive spots from infected *S*. *mansoni* sera were numbered (n = 23) and were excised and submitted for mass spectrometry identification.

In native 2D-WB (Fig 1B), 23 immunoreactive spots were recognized by the pooled infected sera from *S*. *mansoni*. No difference in recognition was seen between chronic and acute sera (groups 1 and 4). From 23 spots, 22 spots were simultaneously recognized by STH-positive sera (group 3) and 10 spots were recognized by negative sera (group 5). One single spot, number 5 (indicated by white arrow on Fig 1), was exclusively recognized by infected patients (acute and chronic) and was not recognized by the STH-infected and health individuals. Spot 5 was detected by antibodies in the pooled 180-day post-treatment sera (group 2). The immunoblot and homologous stained gel were aligned, and the 23 spots matched and excised for LC/MS analysis (Fig 2). The identification of spots is presented in Table 1.

**Table 1:** Identities of proteins recognized by *S*. *mansoni*-positive serum (acute and chronic individuals) in soluble egg extracts.

Spot	Accession	Description	Unique peptides	Coverage (%)	MW (kDa)	pI	Score	Biological function
1	Smp_170410.1	NADH: ubiquinone oxidoreductase complex I	7	25.83	29,2	4.7	37.83	EM
2	Smp_049550.1	78-kDa glucose regulated protein	17	32.87	71,2	5.22	132.4	EM
3	Smp_106930.1	Heat shock 70 kDa protein homolog	24	48.04	69,8	5.58	229.93	CH
4	Smp_183710.1	Actin	4	44.15	41,7	5.48	151.51	ST
**5**	**Smp_049250.1**	**Major egg antigen**	14	48.20	37,0	6.96	364.77	CH
6	Smp_005880.1	Phosphoenolpyruvate carboxykinase	27	50.96	70,3	6.90	114.13	EM
7	Smp_056970.1	Glyceraldehyde-3-phosphate dehydrogenase	3	10.36	36,4	8.05	7.89	EM
8	Smp_056970.1	Glyceraldehyde-3-phosphate dehydrogenase	9	30.18	36,4	8.05	22.18	EM
9	Smp_056970.1	Glyceraldehyde-3-phosphate dehydrogenase	12	37.87	36,4	8.05	33.84	EM
10	No significant score*	---	---	---	---	---	---	---
11	Smp_150240.1	Secretory glycoprotein k5	3	3.76	31,1	8.19	6.67	ST
12	Smp_079920.1	Pyruvate dehydrogenase	2	5.34	44,0	8.46	1.73	EM
13	Smp_042160.1	Fructose-bisphosphate aldolase	8	23.15	35,4	8.06	27.89	EM
14	No significant score*	---	---	---	---	---	---	---
15	Smp_179250.1	Alpha galactosidase: alpha n	15	30.84	108,4	7.37	105.84	EM
16	Smp_179250.1	Alpha galactosidase: alpha n	2	22.93	47,7	7.53	42.76	EM
17	Smp_150240.1	Secretory glycoprotein k5	3	9.40	31,1	8.19	25.98	ST
18	Smp_035270.1	Cytosolic malate dehydrogenase	12	36.27	31,3	8.48	55.54	EM
19	No significant score*	---	---	---	---	---	---	---
20	No significant score*	---	---	---	---	---	---	---
21	No significant score*	---	---	---	---	---	---	---
22	Smp_150240.1	Secretory glycoprotein k5	3	15.41	31,1	8.19	41.09	ST
23	Smp_150240.1	Secretory glycoprotein k5	2	9.02	31,1	8.19	23.59	ST

* No significant score based on SEQUEST HT search engine output statistic. EM: Energetic metabolism. CH: Chaperoning. ST: Structure/motor activity. All *Smp* IDs can be found in genedb.org. In bold: protein selected to in vitro recombinant protein expression. Spot numbers are related to the Fig 2.

The 23 spots were resolved into 12 proteins. LC/MS analysis revealed instances in which different spots were derived from the same protein: for example, spots 11, 17, 22, and 23 are all secretory glycoprotein k5. It was observed that, in some cases, there was no direct correlation between the amount of protein in the SEE protein extract and its antigenicity level. Although most of the immunoreactive spots recognized by infected serum were visible in the corresponding 2D-PAGE, there were highly immunoreactive spots that were barely visible in stained gels (e.g., spot 1). Spots 10, 19, 20 and 21 were not identified due to low abundance. Most identified proteins were related to housekeeping proteins. These include structural/muscle proteins, enzymes (mostly components of the glycolytic pathway) and chaperone proteins.

To evaluate the presence of glycosylated epitopes on the 23 immunoreactive spots, 2D-WB was performed using SMP-treated membranes (Fig 1C) and then compared to the native one (Table 2). After oxidation, only 12/23 spots maintained immunoreactivity, indicating they potentially have protein epitopes. From these 12 spots, 11 spots cross-reacted with the STH-positive sera (group 3) and 10 spots cross-reacted with negative sera (group 5). Spot number 5 was uniquely recognized by *S*. *mansoni*-infected groups in chronic and acute phase (group 1 and 4) and was not recognized in uninfected groups (group 3 and 5). Furthermore, there was an observed decrease in immune recognition of spot 5 in 180-day post-treatment sera (group 2) compared to the corresponding chronic sera at baseline (group 1) in the SMP experiment.

**Table 2:** Comparative spot recognition by *S*. *mansoni*-positive and negative serum in soluble egg extract before and after sodium metaperiodate oxidation.

Spot	Description	Native 2D-WB					SMP 2D-WB				
		G1	G2	G3	G4	G5	G1	G2	G3	G4	G5
1	NADH: ubiquinone oxidoreductase complex I	X	X	X	X						
2	78-kDa glucose regulated protein	X	X	X	X		X	X	X	X	
3	Heat shock 70 kDa protein homolog	X	X	X	X	X	X	X	X	X	X
4	Actin	X	X	X	X						
5	Major egg antigen*	X	X		X		X	X		X	
6	Phosphoenolpyruvate carboxykinase	X	X	X	X	X	X	X	X	X	X
7	Glyceraldehyde-3-phosphate dehydrogenase	X	X	X	X	X	X	X	X	X	X
8	Glyceraldehyde-3-phosphate dehydrogenase	X	X	X	X	X	X	X	X	X	X
9	Glyceraldehyde-3-phosphate dehydrogenase	X	X	X	X	X	X	X	X	X	X
10	---	X	X	X	X	X	X	X	X	X	X
11	Secretory glycoprotein k5	X	X	X	X	X	X	X	X	X	X
12	Pyruvate dehydrogenase	X	X	X	X	X	X	X	X	X	X
13	Fructose-bisphosphate aldolase	X	X	X	X	X	X	X	X	X	X
14	Fructose-bisphosphate aldolase	X	X	X	X	X	X	X	X	X	X
15	Alpha galactosidase: alpha n	X	X	X	X						
16	Alpha galactosidase: alpha n	X	X	X	X						
17	Secretory glycoprotein k5	X	X	X	X						
18	Cytosolic malate dehydrogenase	X	X	X	X						
19	---	X	X	X	X						
20	---	X	X	X	X						
21	---	X	X	X	X						
22	Secretory glycoprotein k5	X	X	X	X						
23	Secretory glycoprotein k5	X	X	X	X						
**No. spots identified by group**		**23**	**23**	**22**	**23**	**10**	**12**	**12**	**11**	**12**	**10**

* protein selected to in vitro recombinant protein expression. G1: *S*. *mansoni* chronic sera; G2: *S*. *mansoni* chronic sera after 180 days post treatment; G3: STH-positive sera (*Ascaris lumbricoides*, *Trichuris trichiura* and *Ancylostoma*); G4: *S*. *mansoni* acute sera, G5: negative sera from health donors. Spot numbers are related to Fig 2.

Spot 5, approximately 40 kDa and pI 7.0, was identified as MEA and chosen for further evaluation in immunodiagnostic assays. Selection was based on: 1) single identification in infected *S*. *mansoni* individuals (group 1 and 4), 2) absence of cross-reaction in *S*. *mansoni* uninfected individuals (group 3 and 5), 3) recognition after SMP treatment (potential presence of immunogenic peptides and feasibility for bacterial production) and 4) apparent decrease of reactivity intensity by 180 days post-treatment (group 2).

### Recombinant MEA maintained the recognition by sera from *S*. *mansoni* individuals

**Fig 3:**
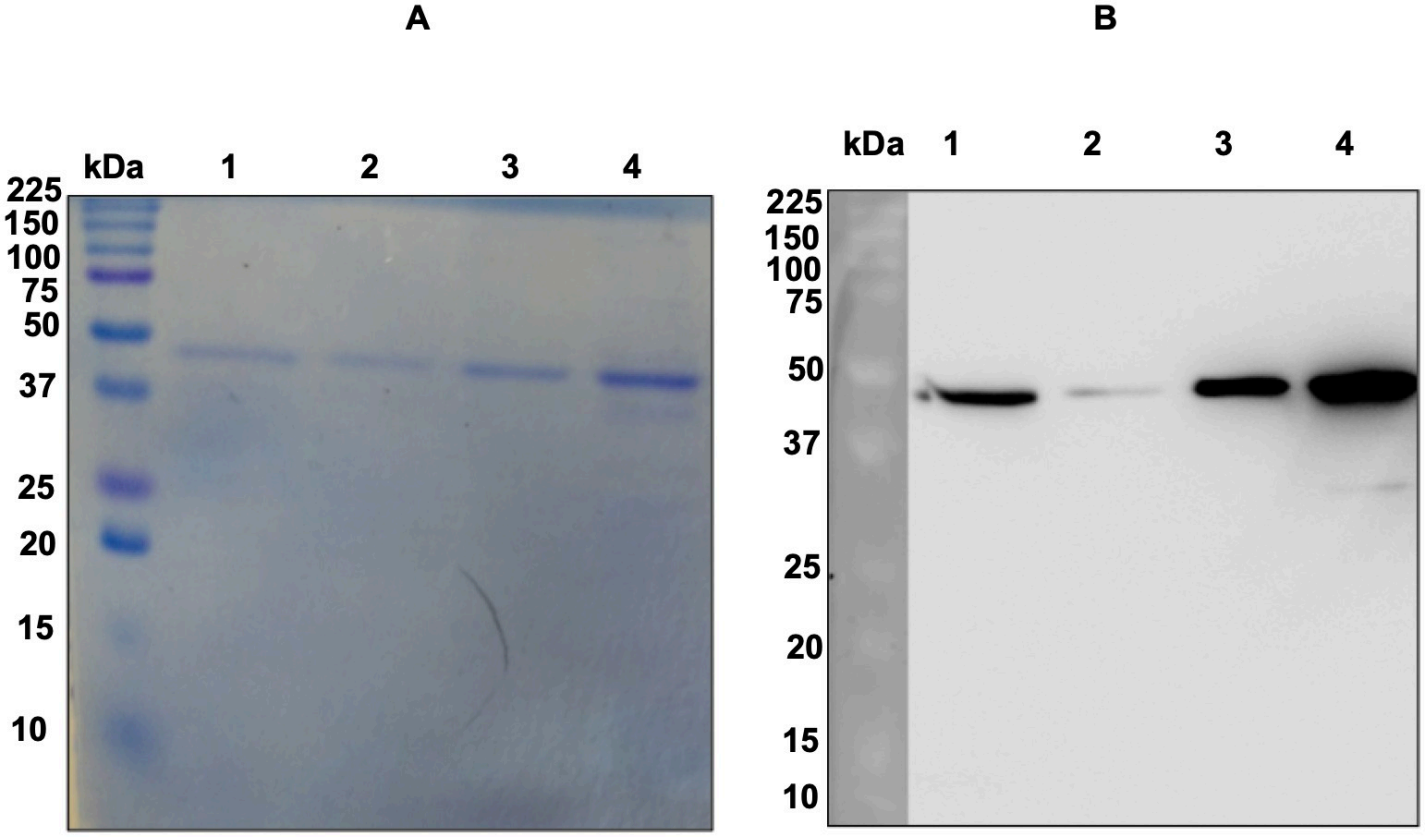
Coomassie blue-stained SDS-PAGE and western blotting anti-his tag with the purified rMEA. Purified rMEA was run in 12% gel (A). Replicate gel was transferred to PVDF membrane and probed with mouse anti-histidine tag followed of anti-IgG conjugated with peroxidase (B).

The rMEA was expressed by IPTG induction in *E*. *coli*. The size from recombinant construction was predicted in Expasy Software including the histidine tag (https://www.expasy.org/proteomics/protein_structure) corresponding to 43 kDa. As shown in Fig 3, the purified protein was present in the gel and the corresponding western blotted anti-histidine tag. To validate the recombinant proteins, the purified material was sent for MS analysis by Shotgun. The results showed 98.6% of abundance was related to native MEA (Smp_049250.1), confirming its identity.

**Fig 4:**
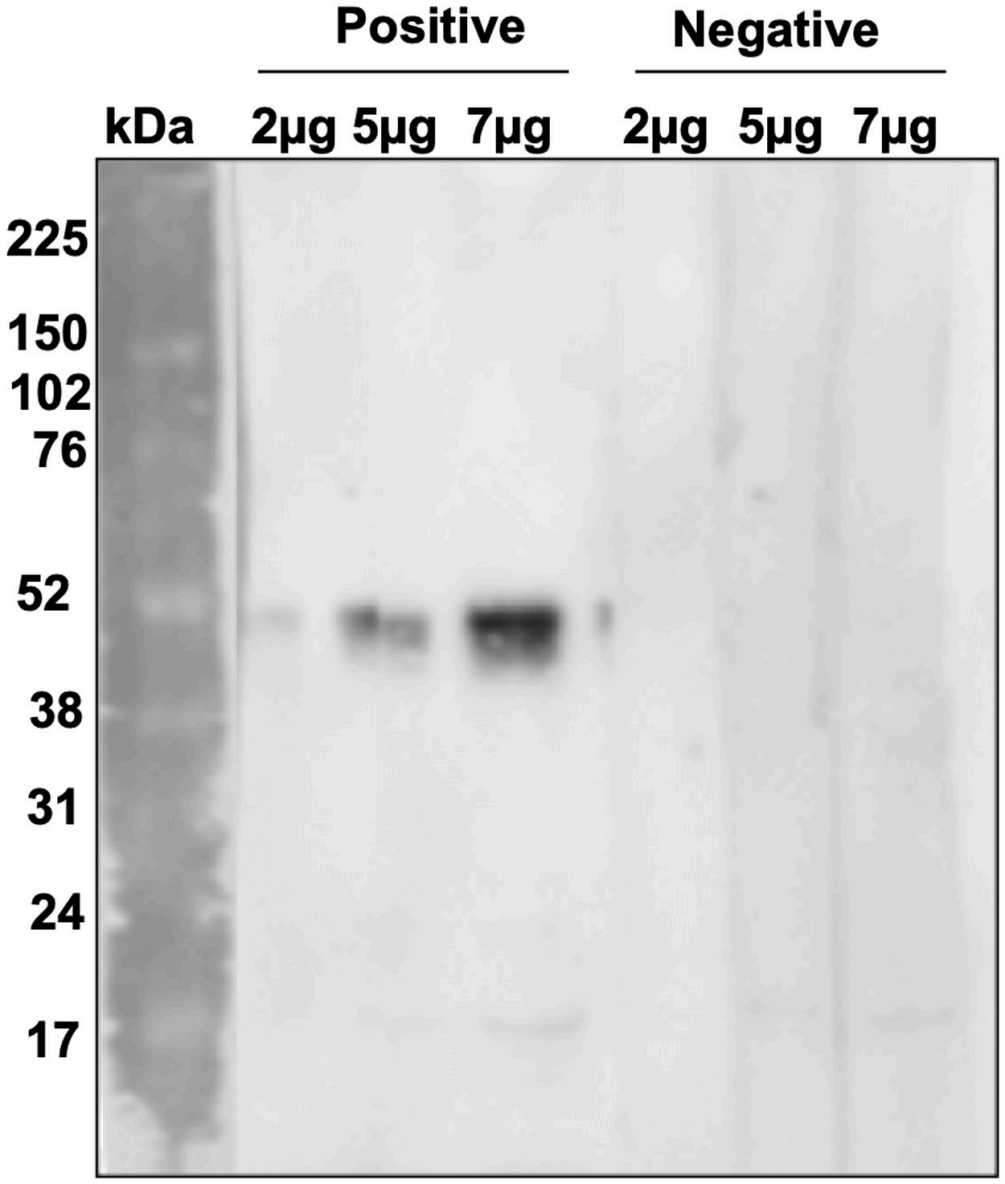
Western blotting anti-rMEA using sera from positive and negative individuals for *S*. *mansoni* infection. A PVDF membrane was coated with three different concentrations of rMEA (2, 5 and 7 μg). The lanes were probed with positive (Group 1) and negative (Group 5) pooled sera at 1:800 dilution. The peroxidase conjugated anti-human IgG antibody was added at 1:80,000. Group 1: *S*. *mansoni* chronic sera; Group 5: negative sera from health donors.

We evaluated the antigenicity from rMEA using serum from *S*. *mansoni* infected individuals from endemic areas and non-infected healthy individuals (NEG). The rMEA maintained the recognition pattern from native form, which was recognized by positive but not negative sera. Also, no unspecific bands were visualized in WB experiment. These data confirmed the correct purification of rMEA and the presence of antigenic epitopes in the *in vitro* prokaryote expression (Fig 4). Once we demonstrated potential for diagnostic application, rMEA was evaluated for detection of IgG by ELISA.

### rMEA-IgG-ELISA was more sensitive than 2 KK slides to detect low-intensity infections

The ROC was carried out to estimate the cut-off and performance indices (sensitivity, specificity, PPV, NPV and AUC) for rMEA-IgG-ELISA. The samples from group 1 (positive from endemic area) (Table 3) and group 5 (negative from health donors) were used as reference. The average intensity of infection of 93 individuals from group 1 was 5.4 EPG, calculated by the geometric mean of the number of EPG **(GMEPG)** in examination of 2 grams of feces.

**Table 3:** Individuals positive for *S*. *mansoni* infection evaluated in IgG-ELISA.

Variables	Category	Total	
		N	%
**Sex**			
	Male	52	55.9
	Female	41	44.1
	**Total**	**93**	**100.0**
**Age**			
	≤ 10	7	7.5
	11–20	29	31.2
	21–40	26	28.0
	41–60	21	22.6
	>60	10	10.8
	**Total**	**93**	**100.0**
**Egg load (EPG)**			
	≤ 10	64	68.8
	11–25	12	12.9
	26–50	3	3.2
	51–99	8	8.6
	≥ 100	6	6.5
	**Total**	**93**	**100.0**

Individuals positive for *S*. *mansoni*, but not infected with other soil-transmitted helminths (Group 1). EPG: eggs per gram of feces.

**Fig 5:**
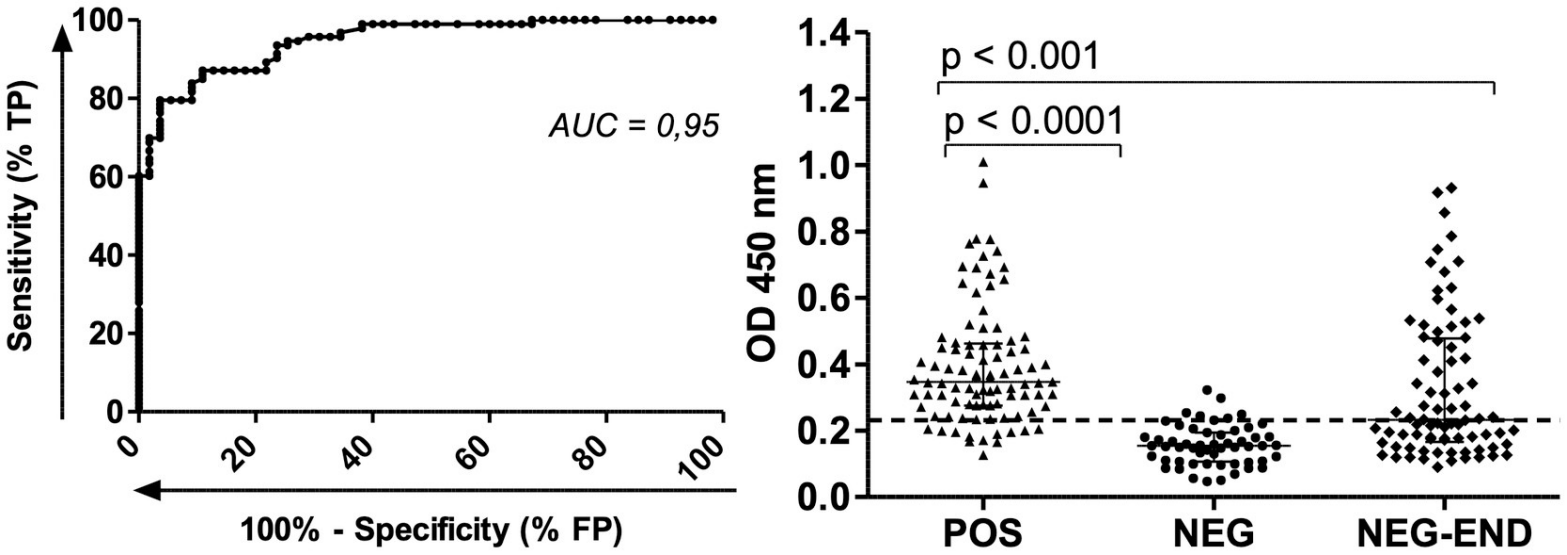
ROC analysis and human IgG-specific response against rMEA. ROC was carried out with 93 positive sera samples from group 1 (POS) and 55 negative sera samples from group 5 (NEG). Additionally, 80 samples from individuals with negative stool examination from endemic areas (NEG-END) were evaluated. Significant differences between groups are indicated in the graphic (Mann Whitney test, CI: 95%). Dashed lines represent the cut-off in the absorbance level, which determines specificity and sensitivity. Group 1: *S*. *mansoni* chronic sera; Group 5: negative sera from health donors.

The AUC demonstrated a high power of discrimination between the groups (AUC = 0.95). The cut-off was 0.232 which was selected based on the best overall accuracy (ACC = 87.8%). Significant IgG reactivity against rMEA was observed in *S*. *mansoni* infected individuals in comparison with negative healthy donors and those negative from endemic areas (Fig 5). The sensitivity was 87.10% and specificity was 89.09% with PPV and NPV of 93.1% and 80.33% respectively. The agreement between rMEA-IgG-ELISA and the reference method determined by 24 slides of KK and 2 procedures of SG showed substantial concordance (κ = 0.75). When the current adopted KK (2 slides) was compared, it demonstrated a fair concordance (κ = 0.32) with a significant difference between the positivity rates (McNemar’s test, p < 0.0001). The positivity rate from rMEA-IgG-ELISA (58.8%, 87/148) and the reference method (62.8%, 93/148) showed no significant difference (McNemar’s test, p = 0.24) (Table 4).

**Table 4:** Performance of rMEA-IgG-ELISA and KK (2 slides) compared with the reference standard (saline gradient and KK 24 slides).

Method	TP	FP	TN	FN	Positivity rate % (n positives / n total)	Sensitivity % (CI 95%)	Specificity % (CI 95%)	PPV (%)	NPV (%)	Kappa (CI 95%)	McNemar test (*p* value)
rMEA-IgG-ELISA	81	6	49	12	58.8 (87/148)	87.1 (78.55–93.15)	89.09 (77.75–95.89)	93.1 (85.59–97.43)	80.33 (68.16–89.40)	0.75 (58.47–90.58)	0.24
KK (2 slides)	36	0	55	57	24.3 (36/148)	38.7 (28.78–49.38)	100 (93.51–100)	100 (90.26–100)	49.11 (39.54–58.73)	0.32 (20.14–43.75)	< 0.0001

The data show the number of true positive (TP), false positive (FP), true negative (TN), and false negative (FN) individuals, as well as the prevalence (%), sensitivity, specificity, kappa index, McNemar *p* value, together with the respective confidence intervals (CI), of concordance of each test.

K-K identified 36/93 positive cases (38.7% sensitivity), yielding 57 false negative results. The rMEA-IgG-ELISA was able to identify 81/93 infections, of which 75 were low-intensity infections (< 100 EPG). From the group harboring extremely low-intensity infections (≤ 10 EPG), the immunoassay identified 56/64 (87.5% sensitivity), of which 27 had intensity of infection at 1 EPG (Table 5). rMEA-IgG-ELISA determined 12 false negative results, which the egg burden varied from 1 to 99 EPG. There was not a significant positive correlation between IgG levels (OD) and egg burden (EPG) by Spearman rank test (r = 0.024, p = 0.8167). From 80 stool negative individuals from the endemic area, rMEA-IgG-ELISA identified 41 as positive.

**Table 5:** Sensitivity of rMEA-IgG-ELISA for the detection of schistosomiasis considering the classification by egg load.

Classification by egg load (EPG)	% Sensitivity (n positive / n total)
Low-intensity (1–99 EPG)	
≤ 10	87.5 (56/64)
11–25	83.3 (10/12)
26–50	66.7 (2/3)
51–99	87.5 (7/8)
Moderate (100–399 EPG)	
≥100	100.0 (6/6)

Egg load defined by egg counts of 2 grams of feces (24 Kato-Katz slides and 2 Saline Gradient). EPG: egg per gram of feces.

## Discussion

Advances in development of new schistosomiasis diagnostic methods are necessary for low prevalence/low-intensity infections [1]. In the majority of Brazilian endemic areas, transmission is maintained by individuals having low level infections that are undiagnosed by analysis of 2 slides of KK in a single stool sample, as recommended by WHO [5, 9]. Despite extensive efforts over several years, the search for sensitive and specific diagnostics for schistosomiasis is ongoing. Diagnosis using antibodies [37, 45, 46], antigens [65, 66] or DNA [8, 16, 67] show high sensitivity, but reduced specificity compared to egg-based methods [7, 14, 48]. Improvements on immunoassays have been the most studied aspect of mapping, due to promising ability to detect individuals with low-intensity infections undiagnosed by standard KK [29, 37, 45, 65, 66, 68–70]. In addition, they have paved the way for less laborious rapid tests that are useful both in communities in endemic areas and at point-of-care facilities [66, 71].

The POC-CCA is the antigen-based method most recently evaluated to be part of WHO guidelines. This immunochromatographic RDT has been commercially available since 2008 and it is based on detection of CCA in urine samples. As this antigen is released into the circulation by adult schistosomes, its detection levels can indicate an active infection [24]. The POC-CCA has shown good performance in Africa and has been proposed as a substitute for the KK method based on its estimated higher sensitivity and operational advantages, especially in highly endemic areas [68]. However, in low-endemic areas, especially in Brazil where extreme low-intensity infections (1–25 EPG) are predominant, the results are controversial and more evaluation is needed before the test is released for general use [6–8, 30]. The issues surrounding adopting POC-CCA in Brazil are related to 1) inadequate estimation of sensitivity and specificity of the POC-CCA, due to the absence of adoption of a highly sensitive method as a reference standard [7, 14, 32–34]; 2) high number of individuals incorrectly diagnosed (false positive and false negative) due to interpretation of trace as positive or negative [6, 14, 29, 31]; 3) the low sensitivity to detect infections with low parasite loads [7, 14, 22]. In a recent study, in order to improve the performance of POC-CCA in low-endemic areas, urine samples were 10-fold concentrated by lyophilization and analysis of 2 grams of feces (24 K-K slides plus 2 SG) served as a reference method. After the additional step, the trace became positive in parasitological positive cases but remained as trace in parasitological negative cases increasing the sensitivity of POC-CCA from 6% to 56% [31]. More validation is going on in order to reduce the time-consuming lyophilization step (34 h) by using 30 kDa-filter centrifugation (50 min), thereby allowing the test to be more suitable for large-scale evaluations [29].

As none of the diagnostic tests used currently provide 100% accuracy, sequential or simultaneous multiple tests are applied to address mapping, monitoring of interventions, assessment of cure rates and disease surveillance [6–8, 14, 15, 17, 71]. Antibody-based immunodiagnostics are particularly useful for detecting low-intensity infections. Since antibodies to the parasite develop during the first weeks after infection, they can be detected before eggs yielding higher sensitivities. Due to greater sensitivity than parasitological methods, these tests allow for detection of infections with loads as low as 1 EPG [22, 36, 37, 40]. The use of antibody detection in low-endemic areas has been successfully applied but it is limited to use as screening tests or as a complementary tool to parasitological evaluation [16, 21, 37–40, 72]. This is due to the inability to accurately differentiate between active infection, past infection, and reinfection; and also because of antibody cross-reactivity with different helminth species [48]. In terms of increasing the specificity of antibody-based immunodiagnostics, the search for new antigens has been proposed [44, 45, 73, 74]. Most of the antigens described in the literature are related to the crude extracts (SEA and SWAP) which have a complex source, require time-consuming purification steps, and vary greatly on accuracy and reproducibility [37, 48]. Molecular cloning and the expression of recombinant proteins represent a reliable alternative for generating enough amounts of well-defined antigens for use in immunodiagnostic assays. For these reasons, the goal of the present study was to identify antigenic targets using immunoproteomic analysis and validate their performance for detection of low burden individuals as an initial step towards development of recombinant protein-based immunodiagnostics.

Our work was the first serological-proteomic study conducted with egg extracts from *S*. *mansoni* and human samples. It included diversified sets of sera allowing for a more rational search for highly specific diagnostic molecules. Through the immunoproteomic approach, we identified 12 different immunogenic proteins from egg extracts. Other *Schistosoma spp*. serological-proteomic studies using human samples have been conducted. Mutapi et al. (2005) used serum from infected individuals with *S*. *hematobium* to screen adult worm antigens in 2D-PAGE to identify suitable antigens for diagnostic purposes. Twenty-six immunoreactive protein spots were identified and investigated [56]. The unique study related to *S*. *mansoni* and human samples involved searching for vaccine candidates using worm extracts. Ludolf et al. (2014) identified 47 different immunoreactive proteins from worm antigens using sera from positive and negative endemic individuals. One of them, the eukaryotic translation elongation factor, uniquely reacted with naturally resistant residents from endemic areas and was considered a potential vaccine candidate [57].

Our results showed that 23 immunoreactive spots, resolved into 12 different proteins, were strongly recognized by pooled sera from *S*. *mansoni*-infected individuals. No differences were found between acute and chronic samples. Currently, differentiation between the two stages of infection is based on clinical and epidemiological data. Differentiating them by serological diagnosis could contribute to the establishment of adequate protocols for treatment of infected patients and detection of new foci or infection cases in tourists. However, this work did not identify proteins specific for different stages of infection. Some studies initially pointed out antigens, such as SmRP26 and KLH, with the potential to discriminate between the acute and chronic phase, however, there was no reproducibility in subsequent evaluations [75–77]. de Assis et al. (2016) evaluated the recognition of 92 proteins in sera from positive (acute and chronic phase) and negative individuals by using protein microarrays. Fifty antigens were recognized by sera samples in the acute and chronic phase. From these, 4 antigens were differentially recognized between the acute and chronic phase and will be further evaluated in the standardization and validation of new differential methods for the diagnosis of different infection stages [78].

Differential recognition was not found between the infected group and post-treatment group. Antibodies remain present in serum following treatment of infected individuals, making it difficult to differentiate between current and previous infections [48]. The persistence of antibodies after treatment impairs post-treatment monitoring, which could be resolved by means of a differential diagnosis using an antigen specific for that phase. Mutapi et al. (2005), using a similar approach to this work, but with *S*. *haematobium* infections, identified 5 exclusively immunoreactive proteins in serological post-treatment samples. The presence of new antigens at this stage was related to the release of these antigens after parasite death and exposure to the host’s immune system [56].

In order to analyze if the antigenicity from proteins was carbohydrate dependent, we screened the extract after SMP treatment. Periodate oxidation alters glycan structures from glycoproteins and therefore eliminates their ability to be detected by anti-glycan antibodies [62]. This finding has strong implications for selection of a more specific target and choice of appropriate vectors to express recombinant candidates for the development of diagnostic tests. Polyparasitism is common in endemic areas and glycans are the most shared and most immunogenic fractions among helminth species [60, 79, 80]. Alarcon de Noya et al. (2000) demonstrated that after oxidation of egg extracts, the specificity from IgG-ELISA in detecting *S*. *mansoni*-infected individuals increased from 73% to 97% due to reduction of cross-reactivity with other parasites [49]. In this study, from 23 spots recognized in native SEE extract by the *S*. *mansoni*-positive group, 22 cross-reacted with STH-positive individuals. After the oxidation step, the number of spots recognized by the *S*. *mansoni*-positive group decreased to 12, indicating the influence of carbohydrate moieties on the antigenicity of proteins. The treatment with SMP did not influence the antigenicity from spot 5 (MEA), the only protein which maintained recognition in the *S*. *mansoni*-positive group and was absent in STH-positive groups. This suggests the presence of protein epitopes and enabled the selection of a prokaryotic system for production of recombinant MEA. Glycoproteins with carbohydrate-dependent antigenicity require eukaryotic expression due their ability to undergo post-translation modifications, such as glycosylation. This system is less attractive for diagnosis purposes as it is more laborious, complex and expensive to adopt [81].

MEA was selected to be evaluated as an immunodiagnostic for schistosomiasis because it was the only antigen that was recognized by *S*. *mansoni*-infected patients, but was not recognized by negative individuals and those infected with other STH in native and SMP evaluations. MEA, also known as Smp40, is one of the 40 most abundant proteins secreted by the eggs [79, 82, 83]. MEA is a chaperone and shares homology with the family of heat shock proteins. It is involved in the protection of miracidia from oxidative stress, denaturation, and aggregation of proteins [79]. In the study by Nene et al. (1986), when western blots were probed with serum raised against a Smp40 fusion protein, the Smp40 could be detected in adults, cercariae, schistosomulum and egg stages [84]. van Balkon et al. (2005) also demonstrated the MEA is present on tegmental and stripped worms protein fractions [85]. In humans, MEA has been described to initiate a strong T-cell response, which is associated with reduced granuloma formation [86, 87].

The potential for diagnostic application of MEA was observed in study by Ludolf et al. (2014). MEA was selected by immunoproteomic analysis of adult worm and its recombinant form demonstrated immunoreactivity against samples from chronic individuals using western blotting [57]. In this study, rMEA was recognized by sera from infected endemic individuals and was not recognized by sera from negative non-endemic individuals in WB analysis. Since this antigen proved to be promising in preliminary WB, we evaluated the performance of rMEA in the detection of antibodies IgG by ELISA.

rMEA-IgG-ELISA performed significantly better than the currently adopted KK (2 slides) for detection of low-intensity infections. When compared to a reference standard (24 KK + 2 SG), the test showed sensitivity of 87.10% and specificity of 89.09% represented by AUC = 0.95. On other hand, the KK performed by 2 slides exhibited a sensitivity of 38.71% and 57 false negative cases. These 57 misdiagnosed individuals were verified have 1–10 EPG, which is indicative of the majority of cases of schistosome infection in Brazil [6–8, 22, 29, 31]. These individuals would not receive treatment, possibly develop serious forms of infection, and contribute to maintenance of transmission. Differently, rMEA-IgG-ELISA identified 56 from 64 cases from group having ≤ 10 EPG, demonstrating its high sensitivity for identifying extreme low intensity infections. Even though POC-CCA has been encouraged for use, as it is based on direct detection, it presents the same low sensitivity as KK (2 slides) to detect low burden infection. In two different studies from Brazil, regardless if traces were considered positive or negative, the POC-CCA sensitivities only ranged from 14–47% compared to a reference standard. Further, around one third of positive individuals misdiagnosed by POC-CCA in these studies had loads 1–10 EPG [7, 14].

As observed here, other studies have demonstrated the ability of ELISA to identify low burden individuals missed by 2 KK analysis in low-endemic areas in Brazil, as well as have demonstrated a good correlation compared to an improved reference method. IgG-ELISA-SWAP showed 90% sensitivity/specificity and Kappa index 0.85 when compared to 18 K-K slides [37]. The study by Oliveira et al. (2005) [40] demonstrated 98% sensitivity and 97.7% specificity for IgM-ELISA. Both studies identified low burden individuals undiagnosed when 1 K-K slide was used. Although crude extract antigens can be used for ELISA, such assays would require infrastructure to maintain the parasite cycle and the complexity of large-scale production and standardization. In respect to use of *S*.*mansoni* recombinant proteins, the previous studies also reported high levels of sensitivity and specificity similar to rMEA for detecting low-intensity infections. The recombinant CCA showed 100% sensitivity and 96% specificity by IgG detection in chronic individuals using magnetic microspheres without false-negative results [47]. The IgG-ELISA using recombinant 200-kDa tegumental protein demonstrated 90% sensitivity and 93.3% specificity with a strong correlation with egg burden in the same set of individuals [74]. El Aswad et al. (2011) showed sensitivity and specificity of 89.7% and 100%, respectively, using the recombinant calreticulin and cercarial transformation fluid in ELISA [88].

The rMEA-IgG-ELISA determined that a number of negative residents from endemic areas were positive. In endemic regions, residents are continuously exposed to parasite infection and parasite antigens; many have high titers of antibodies without being infected, leading to a large-number of false positive results [37]. The false positive issue has been reported in other studies discussing single test immunoassay and why a single immunodiagnostic assay may not be appropriate for epidemiological surveys [58, 88–90]. Even though the high performance of immunoassays indicates them as alternatives to the standardized K-K in terms of preventing false negative results, the presence of false positives can yield significant over-treatment, making them not optimized for single use tests. On the other hand, combined approaches have been successful in diagnostic screening, whereby individuals are initially tested for the presence of antischistosomal antibodies and then those with positive results are confirmed by copro-microscopy techniques. In Brazil, this combination has led to accurate diagnoses and help inform treatment decisions [16, 17, 21].

In the work presented here, we demonstrated that the immunoproteomic approach was successful in selecting a good candidate for use in the diagnosis of schistosomiasis, as confirmed by 2D-PAGE and western blotting analysis. Although rMEA was capable of detecting low parasite burden infections that were undiagnosed by 2 slides of K-K, the sensitivity and specificity were 87.10% and 89.09%, respectively, and there was not a significant correlation between the IgG absorbance and the egg burden. Our results indicate that the use of MEA in indirect immunoassays can be valuable when used as a screening tool during epidemiological surveys, followed by more specific assays for a robust parasitological evaluation. To overcome the complexity of ELISA in the field, a second-generation of antibody-based RDTs has already been proposed, as well as the detection of antigen together in a multiplex strip on a reader [66]. Accordingly, new RDTs platforms should take better advantage of antibodies for the specific detection of protein epitopes to be an alternative method to distinguish active infections.
